# Heterologous Expression of Pseudouridimycin and Description of the Corresponding Minimal Biosynthetic Gene Cluster

**DOI:** 10.3390/molecules26020510

**Published:** 2021-01-19

**Authors:** Nils Böhringer, Maria A. Patras, Till F. Schäberle

**Affiliations:** 1 Institute for Insect Biotechnology, Justus-Liebig-University of Giessen, 35392 Giessen, Germany; nils.boehringer@agrar.uni-giessen.de; 2 German Center for Infection Research (DZIF), Partner Site Giessen-Marburg-Langen, 35392 Giessen, Germany; 3 Fraunhofer Institute for Molecular Biology and Applied Ecology (IME), Branch for Bioresources, 35392 Giessen, Germany; Maria.Patras@ime.fraunhofer.de

**Keywords:** antibiotic, natural product, pseudouridimycin, PUM, heterologous expression

## Abstract

Pseudouridimycin (PUM) was recently discovered from *Streptomyces* sp. DSM26212 as a novel bacterial nucleoside analog that competes with UTP for access to the RNA polymerase (RNAP) active site, thereby inhibiting bacterial RNAP by blocking transcription. This represents a novel antibacterial mode of action and it is known that PUM inhibits bacterial RNAP in vitro, inhibits bacterial growth in vitro, and was active in vivo in a mouse infection model of *Streptococcus pyogenes* peritonitis. The biosynthetic gene cluster (BGC) was previously identified and characterized by knockout experiments. However, the minimal set of genes necessary for PUM production was not proposed. To identify the minimal BGC and to create a plug-and-play production platform for PUM and its biosynthetic precursors, several versions of a redesigned PUM BGC were generated and expressed in the heterologous host *Streptomyces coelicolor* M1146 under control of strong promotors. Heterologous expression allowed identification of the putative serine/threonine kinase PumF as an enzyme essential for heterologous PUM production and thus corroboration of the PUM minimal BGC.

## 1. Introduction

Pseudouridimycin (PUM) is a novel bacterial RNA polymerase (RNAP) inhibitor that was discovered in *Streptomyces* sp. DSM26212 and characterized by Maffioli et al. in 2017 [1]. The DNA-dependent bacterial RNAP is a target for antibiotics that is of proven interest for antibiotic drug development. Known inhibitors are the rifamycins, e.g., rifampicin that is applied in treatment of tuberculosis, leprosy, and AIDS-associated mycobacterial infections [2,3]; the lipiarmycins, e.g., the narrow-spectrum antibiotic fidaxomicin used for treatment of *Clostridium difficile*-related infections [3]; α-pyrone antibiotics, e.g., corallopyronin and myxopyronin, both currently under preclinical evaluation [4,5,6,7,8]. All these antibiotics block transcription by binding to RNAP, which results either in a steric blocking of nascent RNA chain extension after the first or second condensation step (i.e., rifamycins), by inhibition of transcription initiation through binding to the DNA template–RNAP complex (i.e., lipiarmycins), or by blocking the hinge region of the RNAP (i.e., α-pyrone antibiotics). PUM in contrast competes with uracil triphosphate (UTP) for access to the active site cavity of the RNAP. Thereby, incorporation of UTP nucleotides to the growing RNA chain is blocked. This represents a novel mode of action, rendering the compound an interesting lead structure for further investigation and development.

The biosynthetic pathway of PUM and a corresponding biosynthetic gene cluster (BGC) was identified and characterized by knockout (KO) experiments [9]. Furthermore, production of the pseudouridine nucleoside antibiotic strepturidin by *Streptomyces albus* DSM40763 was recently reinvestigated and it could be shown that strepturidin is actually PUM. However, the structural assignment in the initial report was found to be not correct [10]. Genetic analysis of *S. albus* DSM40763 also revealed the presence of the PUM BGC in this strain [10]. For manufacture of PUM, nine catalytic steps were proposed [9]. Thereby, in a first step, uridine nucleotides are converted to pseudouridine nucleotides and subsequently become dephosphorylated by the catalytic action of the pseudouridine synthase PumJ and the phosphatase PumD to yield free pseudouridine (PU). Via intermediate creation of 5′-oxo-PU and subsequent transamination, 5′-amino-PU (APU) is formed by PumI, catalyzing oxidation of the 5′ hydroxyl group to a keto group, and PumG, catalyzing transamination at the 5′ position with asparagine as NH_3_ donor. Then, APU is linked to glutamine by PumK, yielding Gln-APU. In parallel, glycine is converted to guanidinoacetic acid (GAA) by transguanylation (PumN catalyzed), which is subsequently fused to Gln-APU by PumM, resulting in formation of deoxy-PUM. The final biosynthetic step is hydroxylation of the peptide bond/amidic nitrogen between Gln and GAA by PumE. The identified BGC consists of 15 genes (*pumA*-*pumO*). For seven of these genes (i.e., *pumE*, *G*, *I*, *J*, *K*, *M*, and *N*), a function could be assigned by experimental data. For five genes (i.e., *pumB*, *C*, *D*, *H*, and *L*), a function was proposed based on sequence homology. Hence, each of the nine proposed steps was at least in silico matched with a corresponding enzyme from the PUM BGC [9]. The gene *pumH* that is situated within the BGC was proposed to function as an adenylate kinase, based on sequence homology. However, no biosynthetic step could be readily assigned, since it could not be matched to one of the proposed biosynthetic steps in PUM biosynthesis [9]. Deleting the gene from the WT producer strain resulted in a significant drop in production but not in a complete abolition of PUM production. Additionally, one regulator and two transporter genes (i.e., *pumB*, *pumL*) could be readily identified based on homology. In the first reports, no function was assigned to the genes *pumA*, *pumF* and *pumO* in PUM biosynthesis. However, the *pumF* analogue *mur33* is present in the muraymycin C1 BGC, the latter an uridine nucleoside itself.

Here, the heterologous expression of the PUM BGC in the commonly used surrogate host *Streptomyces coelicolor* M1146 is described. Therefore, the BGC was streamlined (i.e., all genes proposed to be essential for the biosynthesis were cloned facing in the same direction and were set under the control of one promoter) and several constructs were generated to identify the minimal BGC necessary for heterologous production of the nucleoside antibiotic. Only the construct harboring, in addition to the described nine biosynthetic steps, the genes *pumH* and *pumF*, the latter showing sequence similarities to genes encoding for a serine/threonine protein kinase, resulted in reliable PUM biosynthesis in the heterologous host applied. Thereby, evidence is presented that the enzyme PumF is essential for PUM biosynthesis by regulating enzymatic activity in the biosynthetic pathway.

## 2. Results

**Figure 1:**
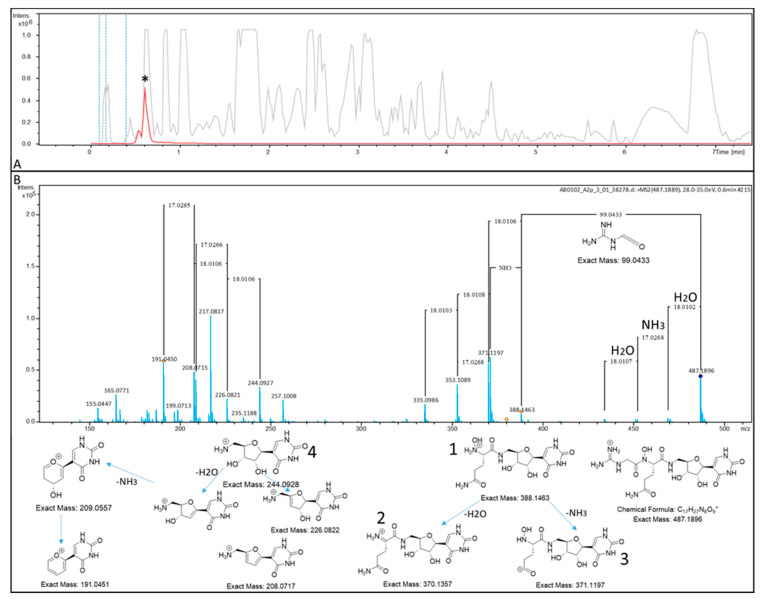
(**A**) High-resolution UHPLC–MS measurement of a PUM-enriched fraction from *Streptomyces* sp. DSM26212. Grey: Base peak chromatogram (BPC). Red: Extracted ion chromatogram (EIC) of C_17_H_26_N_8_O_9_ [M + H]^+^ ± 0.0005. The PUM peak in the EIC is marked with an asterisk (*). (**B**) Fragmentation pattern analysis of the parent ion *m*/*z* 487.1896, corresponding to PUM. Top: Detected MS/MS fragmentation pattern. Bottom: Plausible MS/MS decay products, matching the recorded fragmentation pattern.

The nucleoside-analog inhibitor PUM represents a highly hydrophilic compound. First, the experimental setup for detection of PUM was tested. Therefore, the reported wild-type producer strain *Streptomyces* sp. DSM26212 was cultivated in production medium (PM medium) for 5 days. The fermentation broth was cleared by centrifugation as well as filtration and PUM was enriched by strong cation-exchange chromatography, as previously described [1]. Subsequent high-resolution liquid chromatography/mass spectrometry (LC–MS) analysis revealed the presence of PUM, ionizing as [M + H]^+^ ion in the enriched fraction. The identity of this ion could be corroborated by MS/MS fragmentation pattern analysis. A plausible decay mechanism yielding the observed fragments was proposed and fragments observed in the experiment could be matched (Figure 1).

**Figure 2:**
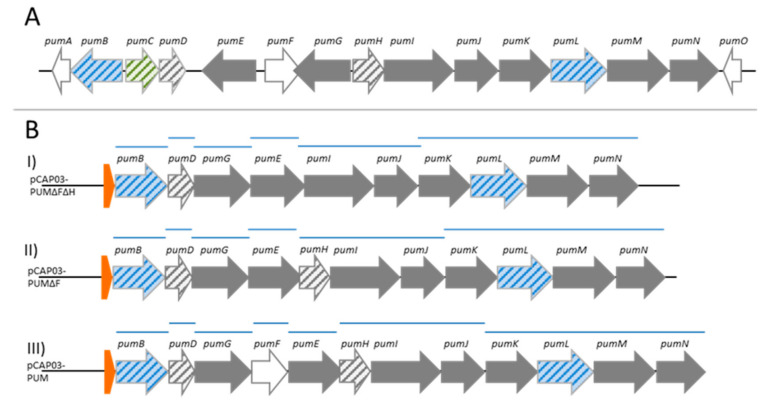
Biosynthetic gene clusters used in this study corresponding to biosynthesis of PUM. (**A**) Native configuration of the PUM BGC in *Streptomyces* sp. DSM26212. (**B**) Rearranged PUM BGCs for heterologous expression in Streptomycetes hosts. All genes are orientated in the same direction and a promoter was added upstream of the first gene *pumB*. Version I (pCAP03-PUMΔFΔH) carries the depicted genes; in version II, *pumH* was added (pCAP03-PUMΔF); and in version III, *pumF* was added (pCAP03-PUM) as well. PCR fragments used for assembly are displayed as blue lines. Blue colored genes correspond to transport-related genes, green corresponds to regulatory elements and white to genes that were not assigned any function in PUM biosynthesis yet [9]. Orange colored elements represent artificial promotors, either constitutive ermE* or inducible tcp830. Genes without experimentally proven function [9] are striped.

To generate a heterologous expression platform for PUM production, a streamlined assembly of the BGC was envisaged, meaning only genes involved in the biosynthesis should be cloned and it was planned to assemble the genes in a way that all face the same direction. However, to avoid autotoxicity, the genes *pumB* and *pumL*, encoding major facilitator superfamily (MFS)-type transporters, which might be essential for self-resistance, should be incorporated into all expression constructs. As a first construct, the previously identified genes to which a function in PUM biosynthesis was assigned (by experimental proof and, in some cases, putatively based on sequence similarity) were amplified from the natural producer strain *Streptomyces* sp. DSM26212 and were cloned into the *E. coli*/*Streptomyces* shuttle vector pCAP03. Hence, the genes *pumB*, *D*, *G*, *E*, *I*–*M* were assembled in the shuttle vector and the resulting construct pCAP03-PUMΔFΔH was outfitted either with the constitutive *Streptomyces* ermE* promotor, or the inducible tcp830 promoter by λRED recombination, whereby the latter has a lower expression strength than ermE* (Figure 2). Subsequently, these constructs were transferred into *S. coelicolor* M1146 [11] by conjugation. Fermentation was performed in complex medium, i.e., in International Streptomyces Project medium 2 (ISP2) and tryptic soy broth (TSB). Thereby, the cultures carrying the tcp830 promoter were induced by addition of anhydrotetracycline (2 mg/L) to the cultures after one day of growth. PUM production was analyzed after 3 and 5 days of fermentation by UHPLC–MS using the cleared culture broth. *S. coelicolor* M1146-pCAP03 (empty vector) served as negative control. However, heterologous expression using the construct pCAP03-PUMΔFΔH (see Figure 2, version I) did not result in detectable PUM production at all. Therefore, it was hypothesized that a lack of correctly phosphorylated uridine precursors prevents detectable PUM production. To verify this assumption, the adenylate kinase encoding *pumH*, which catalyzes the phosphorylation of a uridine-based substrate, was added to the construct, resulting in pCAP03-PUMΔF (Figure 2, version II). Heterologous expression using the latter construct enabled detection of minor traces of an ion corresponding to the calculated mass/charge ratio of the PUM [M + H]^+^ adduct. Such an ion was not present in the negative control. This result pointed towards the fact that this could be regarded as the minimal gene set necessary for production of PUM. However, it was questionable whether this very low expression yield justifies this as the minimal BGC. 

**Figure 3:**
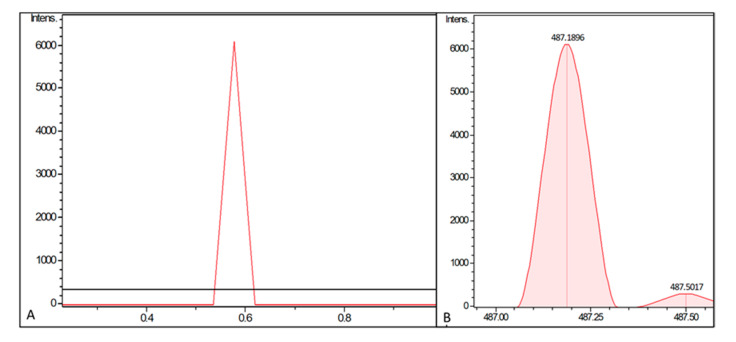
LC–MS analysis of *Streptomyces coelicolor* M1146 fermentation broth. Displayed are (**A**) the extracted ion chromatograms (EICs) for C_17_H_26_N_8_O_9_ [M + H]^+^ ±0.0005 corresponding to PUM. The strain extracts of *S. coelicolor* M1146 + pCAP03 (empty vector control in black) and *S. coelicolor* M1146 + pCAP03-PUM_ermE* (carrying the expression construct version III in red) are shown. Fermentation was performed in ISP2 medium. (**B**) Measured *m*/*z* of the peak, fitting exactly to the calculated value for PUM ions (i.e., 487.1896 *m*/*z*).

It was next determined whether a further gene is essential to enable a valid PUM biosynthesis. A promising candidate was *pumF*, which is located right in the middle of the BGC. BLASTp of the translated nucleotide sequence revealed homology to the *mur33*-encoded protein (77% query coverage; 43% identity) in the BGC coding for muraymycin C1—the latter another nucleoside natural product. BLASTp search of *pumF* and *mur33* showed homology (48% and 50%) to predicted serine/threonine protein kinases of Actinomycetes. Based on these observations, the *pumF* gene was incorporated into the expression construct pCAP03-PUM (Figure 2, version III). Fermentation of an heterologous host carrying the expression construct pCAP03-PUM_ermE*, in which the PUM genes are under control of the ermE* promotor, in ISP2 medium led to detection of a low intensity ion with *m*/*z* 487.1856 after 5 days incubation time. This matched the PUM sum formula of C_17_H_26_N_8_O_9_ [M + H]^+^ (8 ppm error) and retention time, and this ion was absent in the empty vector control. Prolonged incubation (20 days) of the strain led to accumulation of this ion and allowed more accurate detection with *m*/*z* 487.1896 (0 ppm error) (Figure 3). Furthermore, targeted MS/MS fragmentation of this ion revealed the presence of the characteristic signature ion of *m*/*z* 244.0920, which is indicative for the APU fragment (3 ppm error). Thus, the identity of the molecule as PUM was verified and the (indirect) involvement of PumF in PUM biosynthesis was shown. However, no ions corresponding to PUM could be detected in cultures grown in TSB medium and in strains that were harboring the expression constructs in which the streamlined BGC was under the control of the tcp830 promotor.

## 3. Discussion

Previously, the biosynthesis of PUM was characterized by generation of KO mutants in the WT producer strain *Streptomyces* sp. DSM26212. Nine catalytic steps were proposed for the formation of PUM and all steps were matched with plausible enzymatic functions encoded within the BGC, proposing the minimal PUM BGC [9]. While the involvement of the *pumH*-encoded protein in PUM biosynthesis was corroborated by the KO experiments, it was also shown that deletion of *pumH* from the WT producer did lead to a drastically reduced production yield but not to abolition of PUM biosynthesis. Thereby, this result pointed towards a rather regulatory function of PumH. It was reasoned that PumH as adenylate kinase alters the phosphorylation pattern of the precursor uridine, which in turn could be processed into pseudouridine. However, when lacking PumH, free pseudouridine could be sourced in small amounts from tRNA turnover derived from primary metabolism, thereby enabling minute production of PUM [9]. This gives evidence that PumH has a gatekeeper function in funneling uridine-based nucleotides (UMP or UDP) as precursor molecules towards the PUM pathway. This is important for the producing organism in order to ensure that the uridine pool designated to transcription and translation is not drained for PUM production. In this way, PumH would decouple PUM biosynthesis from the primary metabolism uridine pool. However, heterologous expression of the identified genes in *S. coelicolor* M1146, a specialized expression host that shows reduced metabolic background (since several BGCs for specialized metabolites were deleted) [11], including and excluding *pumH* did not lead to clearly detectable production of PUM (i.e., only constructs including *pumH* resulted in traces of PUM at the detection limit). This showed that heterologous production was not only bottlenecked by absence of PumH and provoked the question whether the proposed biosynthetic route covers all steps, hence whether the proposed minimal PUM BGC is correct or whether further important functions are encoded in the BGC.

Further investigation aimed towards identification of a gene, vital for heterologous manufacture of PUM. Here, *pumF* could be identified as the most-promising candidate: the *pumF* gene is located right in the middle of the BGC and shows homology to the gene *mur33* (77% query coverage; 43% identity) in the BGC coding for the nucleoside muraymycin C1. Furthermore, both encoded proteins show homology to predicted serine/threonine protein kinases of Actinomycetes. Enzyme phosphorylation is one of the most common post-translational modifications, often involved in regulation of enzyme activity, i.e., activating or inactivating a given enzyme [12]. Hence, it seemed reasonable to assume that this gene is indirectly involved in the PUM biosynthesis, by regulating the activity of one or more enzymes participating in assembly of the molecule. Heterologous expression of the previously identified minimal BGC incorporating *pumF* led to detectable PUM formation. Thereby, involvement of PumF in the PUM biosynthesis was corroborated.

It has to be emphasized that the presented results do not contradict the initially proposed biosynthetic route [9]; instead, heterologous expression is a further proof of the PUM BGC. However, the collected data here clearly show that the genes *pumH* and *pumF* are vital for heterologous PUM production and hence should be considered components of the minimal BGC necessary for PUM biosynthesis as well. Based on the obtained results, we propose that the genes *pumD*, *E*, *G*, *I*, *J*, *K*, *M*, and *N* code for enzymes directly participating in de novo formation of PUM, while *pumH* serves as a decoupling mechanism of PUM biosynthesis from primary metabolism, as demonstrated before by KO experiments [9]. It seems that the function of PumH is provision of phosphorylated uridine nucleosides that can serve as a substrate for the pseudouridine synthase PumJ. However, the exact biochemistry catalyzed by PumH requires in-depth investigation in future studies. The nature of the ’correct’ phosphorylation pattern remains enigmatic to a certain degree, since UTP, UDP and UMP (analogous to ATP, ADP and AMP) naturally occur in primary metabolism, which PUM biosynthesis is decoupled from by PumH. This might be considered a hint towards cryptic nucleoside phosphorylation for precursor molecules, similar to biosynthesis of the uridine nucleoside analogue natural products nikkomycin Z and polyoxin D [13].

The *pumF* gene encodes a regulatory protein controlling the enzymatic activity of one (or more) of the participating enzymes by post-translational phosphorylation. Due to the presence of minor traces of an ion consistent with PUM, it remains possible that the target enzyme of PumF can retain (poor) activity even in the wrong phosphorylation state or that minor amounts of protein undergo phosphorylation from other serine/threonine kinases from the surrogate hosts genetic background with certain promiscuity.

Despite the proof that the presence of *pumF* is important for PUM biosynthesis, the precise function of PumF remains to be elucidated in future studies, since currently the kinase activity is not proven and a protein target is unknown. Furthermore, heterologous production of PUM was achieved; however, the yields were too low to use this strain as a plug-and-play production platform for PUM and its biosynthetic precursors. Therefore, it would be a long way to optimize production in heterologous strains and future work should continue to focus on WT producer strains, which are also genetically accessible and could be optimized in untargeted ways (i.e., random mutagenesis) and by targeted genetic engineering.

## 4. Materials and Methods 

### 4.1. Cultivation of Bacteria 

*Escherichia coli* for cloning purposes were grown in lysogenic broth (LB) broth or agar supplemented with appropriate antibiotics in 1:1000 dilution from the stock solutions at 37 °C with exception of *E. coli* BW25113 + pKD46, which was grown in Super-Optimal Broth (SOB) medium at 30 °C. Cell growth was monitored by measuring the OD_600_ using an Eppendorf BioSpectrometer in single-use cuvettes with a 10 mm light path. *E. coli* precultures were grown in 5 mL LB in reaction tubes supplemented with appropriate antibiotics. Larger-volume cultivations took place in 100 or 300 mL Erlenmeyer flasks. *E. coli* cryocultures were prepared by mixing 1 mL of an overnight culture of *E. coli* with sterile glycerol and were stored at −80 °C. Streptomycetal strains were cultivated and selected on MS agar plates containing appropriate antibiotics. For isolation of genomic DNA and as preculture, *Streptomyces* strains were grown in ISP2 medium for 2–3 days. Streptomycetes were stored as spore suspension in 20% glycerol at −80 °C. Spores were collected from Mannitol Soy (MS) agar plates as described in Practical *Streptomyces* Engineering [14]. 

### 4.2. UHPLC–MS Analysis of PUM 

For the analysis of PUM, a microTOFq II (Bruker) ESI-qTOF-HRMS mass spectrometer (Bruker Daltonics, Bremen, Germany) coupled to an Agilent 1290 UPLC system with an Acquity UPLC BEH C18 1.7 µm (2.1×100 mm) and Acquity UPLC BEH C18 1.7 µm VanGuard Pre-Column (2.1×5 mm) column setup was used. The UPLC system was run using a gradient (A:H_2_O, 0.1% FA; B: MeCN, 0.1% FA; Flow: 600 µL/min): 0 min: 95%A; 0.30 min: 95%A; 18.00 min: 4.75%A; 18.10 min: 0%A; 22.50 min: 0%A; 22.60 min: 95%A; 25.00 min: 95%A. The column oven was set to 45 °C and the injection volume was set to 5 µL. Data were analyzed using the Bruker DataAnalysis 4.0 software package. Samples were prepared by clearing the supernatant through centrifugation at 20000 rcf and supernatants were directly subjected to the LC–MS analysis. Analysis of LC–MS data did not involve statistical methods.

### 4.3. Sequence Homology Analysis

Sequence homology analysis was performed using the “Basic Local Alignment Tool” (BLAST) [15]. Protein sequences were compared pairwise or to protein sequences deposited in the NCBI database.

### 4.4. General Molecular Biology Techniques

Plasmid DNA was isolated using the innuPREP plasmid mini kit 2.0 (AnalytikJena, Jena, Germany) according to protocol. Genomic DNA was extracted using the innuPREP bacteriaDNA kit (AnalytikJena, Jena, Germany). PCR amplification for cloning purposes was performed using Q5 DNA polymerase (NEB Biolabs, New Brunswick, USA). Test PCRs were performed using GoTAQ (Promega, Madison, USA). Plasmid restriction analysis was performed using standard techniques and NEB enzymes, and DNA fragments were analyzed on 1% or 2% TAE-Agarose gels using MidoriGreen as loading dye and stain and a UV-free blue light transilluminator. GeneRuler 1 kb Plus was used as marker. DNA for cloning purposes was excised from the gel and purified using the Zymoclean large fragment DNA recovery kit according to the manufacturer’s instruction. DNA concentrations were determined with an Eppendorf BioSpectrometer using a 1 mm light path cuvette. *E. coli* were transformed with DNA using the standard electroporation methodology. Subsequently, transformants were plated on LB agar plates containing appropriate antibiotics. A complete list of primers and bacterial strains used in this study can be found in the supporting information (Tables S1 and S2). 

### 4.5. Construction of pGEM-teasy_Apra-ermE* and pGEM-teasy_Apra-tcp830

Promotor sequences of ermE* and tcp830 were obtained from Siegl et al. [16] and Dangel et al. [17] and ordered as primers with overlaps to the apramycin antibiotic resistance cassette of pIJ773 [17] and an artificial RBS behind the promotor region. The plasmid vector pIJ773 was isolated and the promotors ermE* and tcp830 were fused to the *aac(3)* apramycin antibiotic resistance cassette by PCR using Q5 as polymerase and pIJ773 as template. The forward primer used was pIJ773cass_f and tcp830, and ermEp1 and ermEp2 served as reverse primers. Due to the size of the ermE* promotor, amplification had to take place in two individual PCR reactions. Therefore, the first segment of the promotor was fused to the pIJ773-derived *aac(3)* apramycin antibiotic resistance cassette by using ermEp1 as reverse primer for the first PCR. The resulting product was subsequently used as template for the second PCR, with ermEp2 as reverse primer. Final constructs were outfitted with an A overhang by incubating the Q5 PCR fragments with GoTaq polymerase. Therefore, the standard reaction mixture without primers was applied at 72 °C for 1 h. Final products were gel purified and ligated into pGEM-t easy, using pGEM-t easy vector systems (Promega) according to the manufacturer´s instructions and introduced into *E. coli* XL1 blue by electroporation. Correct fusion of the promotors was corroborated by sequencing.

### 4.6. Construction of the Integrative Streptomyces PUM Expression Plasmids pCAP03-PUMΔFΔH_ermE*/tcp830, pCAP03-PUMΔF_ermE*/tcp830 and pCAP03-PUM_ermE*/tcp830 

*Streptomyces* sp. DSM26212 was grown in 30 mL ISP2 medium for 3 days and genomic DNA was extracted; *E. coli* Top10 + pCAP03 was grown in 5 mL LB_kan_ overnight and the plasmid was isolated. The PUM BGC was amplified in several parts with primer pairs pumB_f/r, pumD_f/r, pumG_f/r, pumE_f/pumEdH_r, pumI_f/pumJ_r and pumK_f/pumN_r for pCAP03-PUMΔFΔH, pumB_f/r, pumD_f/r, pumG_f/r, pumE_f/r, pumH_f/pumJ_r and pumK_f/pumN_r for pCAP03-PUMΔF and pumB_f/r, pumD_f/r, pumG_f/pumG+F_r, pumE_f/pumE_r, pumF_f/r, pumH_f/pumJ_r and pumK_f/pumN_r for pCAP03-PUM, respectively, using isolated genomic DNA as a template. pCAP03 was amplified in two parts using the primer pairs pCAP03_1f/r and pCAP03_2f/r and linearized (*Nde*I, *Xho*I) pCAP03 as a template. DNA fragments were gel purified and fused by isothermal assembly using self-made isothermal assembly master mix. In brief, 15 µL of isothermal assembly master mix was thawed on ice and equimolar concentrations of all fragments were mixed in 5 µL and added to the master mix. The reaction mixture was incubated at 50 °C for 1 h and subsequently dialyzed by drop dialysis using MF-Millipore VSWP membranes. Subsequently, *E. coli* Top 10 electrocompetent cells were transformed with 2 µL of dialyzed reaction mixture and correct assembly of pCAP03-PUM pCAP03-PUMΔHFΔH, pCAP03-PUMΔf and pCAP03-PUM was corroborated by restriction analysis using double digests with *Hin*dIII and *Not*I and *Hin*dIII and *Nco*I, respectively. *E. coli* BW25113 + pKD46 was transformed with pCAP03-PUMΔH and pCAP03-PUM and selected on LB_Carb/Kan_ agar. Apramycin resistance cassette/promotor fusions were amplified with the primers Rec_uni_f as forward primer and PUM_ermE*_rec and PUM_tcp830_rec as reverse primer, respectively. Thereby, 40 nucleotide homologous overhangs for introduction of promotors into the construct were added. The PCR reaction was directly purified from the mixture using the ZymoResearch large fragment DNA recovery kit and eluted in 10 µL of H_2_O. *E. coli* BW25113 + pKD46 + pCAP03-PUM was grown in 20 mL SOB supplemented with Carb and Kan for maintenance of the plasmids and 0.1% Arabinose for induction of the λRED genes at 30 °C to an OD600 of ~ 0.5. Cells were harvested, washed twice with ice-cold 10% glycerol and resuspended in the return flow. Subsequently, cells were transformed with 4 µL of the previously recovered PCR fragment, taken up in 800 µL of SOB and left to recover at 37 °C. After recovery, cells were plated on LB_Apra_ and incubated over night at 37 °C to induce loss of the plasmid pKD46. The correct integration was corroborated by colony PCR using GoTaq and the primers Pum_screen, binding to *pumB* and ermE*_rectest and tcp830_rectest respectively, binding to the integrated promotor regions. Conjugation to *S. coelicolor* M1146 was performed by passage of the plasmid through *E. coli* ET 12567 and subsequent tri-parental conjugation, as described in Practical *Streptomyces* Engineering [14].

### 4.7. Heterologous Expression of the PUM BGC 

Precultures of transgenic *Streptomyces* carrying PUM expression plasmids pCAP03-PUMΔH_ermE*/tcp830 and pCAP03-PUM_ermE*/tcp830, respectively, as well as empty vector negative control were grown in Erlenmeyer flasks containing 20 mL ISP2 medium in the absence of antibiotics for two days at 200 rpm and 30 °C. For investigation of PUM production in liquid medium, 100 mL cultures in ISP2 and TSB were inoculated with 1% v/v from the precultures and grown for 5 days in Erlenmeyer flasks at 30 °C and 200 rpm. For the induction of the tcp830 promotor, anhydrotetracycline was added to a final concentration of 2 mg/L after one day. The cultures were grown for 3–5 days and PUM production was analyzed by LC–MS/MS directly from the culture. For *S. coelicolor* M1146 + pCAP03-PUM_ermE*, cultivation was extended to 20 days. Two transconjugants were picked independently from the respective conjugation plates (containing constructs with either ermE* or tcp830 promoter) and were fermented and analyzed by LC–MS. Hence, expressions of the PUM BGC in the heterologous host *S. coelicolor* M1146 were performed in both media tested as biological duplicates. 

## Supplementary Materials

The following are available online, Figure S1: Cloning procedure for construction of pCAP03-PUM and pCAP03-PUM promotor derivatives and subsequent conjugation to *S. coelicolor* M1146. Figure S2: Test restriction of pCAP03-PUMΔHΔF. Figure S3: Test restriction of pCAP03-PUM. Table S1: Strains used in this study. Table S2: Primers used in this study.

## Sample Availability

Digital data and physical copies of plasmids and strains are available from the authors.
