# Supplementary material for: Heterologous Expression of Pseudouridimycin and Description of the Corresponding Minimal Biosynthetic Gene Cluster
Source: Molecules. 2021 Jan 19;26(2):510. doi: 10.3390/molecules26020510 (PMC7835738; doi:10.3390/molecules26020510)
Supplement: Supplementary file 1 [file molecules-26-00510-s001.pdf]

Supporting Information

Heterologous expression of pseudouridimycin and description of the corresponding minimal biosynthetic gene cluster

Nils Böhringer [1,2], Maria A. Patras[3] and Till F. Schäberle [1,2,3,*]

[1] Institute for Insect Biotechnology, Justus-Liebig-University of Giessen, 35392 Giessen, Germany; nils.boehringer@agrar.uni-giessen.de
[2] German Center for Infection Research (DZIF), Partner Site Giessen-Marburg-Langen, Giessen, Germany
[3] Fraunhofer Institute for Molecular Biology and Applied Ecology (IME), Branch for Bioresources, 35392 Giessen, Germany; Maria.Patras@ime.fraunhofer.de
* Correspondence: till.f.schaeberle@agrar.uni-giessen.de

Content

Figure S1: Cloning procedure for construction of pCAP03-PUM and pCAP03-PUM promotor derivatives and subsequent conjugation to *S. coelicolor* M1146.

Figure S2: Test restriction of pCAP03-PUMΔHΔF

Figure S3: Test restriction of pCAP03-PUM

Table S1: Strains used in this study

Table S2: Primers used in this study

Figure S1: Cloning procedure for construction of pCAP03-PUM and pCAP03-PUM promotor derivatives and subsequent conjugation to *S. coelicolor* M1146.

Exemplified for pCAP03-PUMΔF_ermE*/tcp830: Result of the PCRs for A) *pumB* (1), *pumD* (2), *pumG* (3), *pumE* (4), *pumH* - *pumJ* (5), *pumK* - *pumL* (6), pCAP03_part1 (8) and pCAP03_part2 (9); B) Test restrictions of 9 colonies of assembled pCAP03-PUMΔF with *Hin*dIII/*Not*I (left) and *Hin*dIII/*Nco*I (right); C) *in silico* simulation of the restriction pattern of pCAP03-PUMΔF with *Hin*dIII/*Not*I (1) and *Hin*dIII/*Nco*I (2); D) Restriction of pGEM-teasy_Apra-ermE* (1) and pGEM-teasy_Apra-tcp830 (2) with *Eco*RI; E) PCRs for the Apramycin resistance cassette – promotor fusions Apra-ermE* (1) and Apra-tcp830 (2); F) Test PCRs for corroboration of correct integration of Apra-ermE* (1) and Apra-tcp830 (2) into pCAP03-PUM; G) Test PCRs to verify the successful transfer of pCAP03-PUM-tcp830 (1) and pCAP03-PUM-ermE* (3) into *S. coelicolor* M1146, with respective positive controls (2,4).

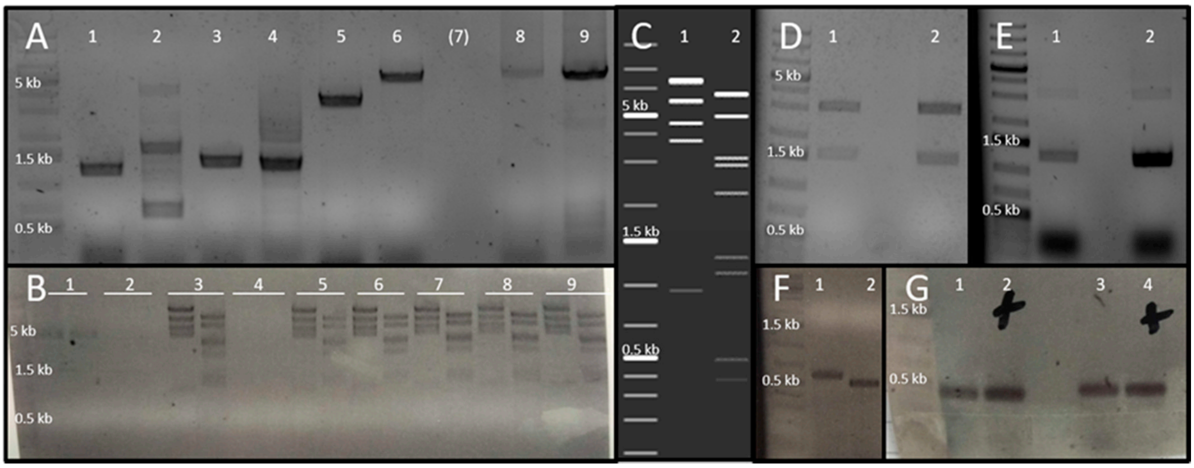

Figure S2: Test restriction of pCAP03-PUMΔHΔF with *Hin*dIII/*Not*I and *Hin*dIII/*Nco*I (left). *In silico* prediction of the restriction analysis (right).

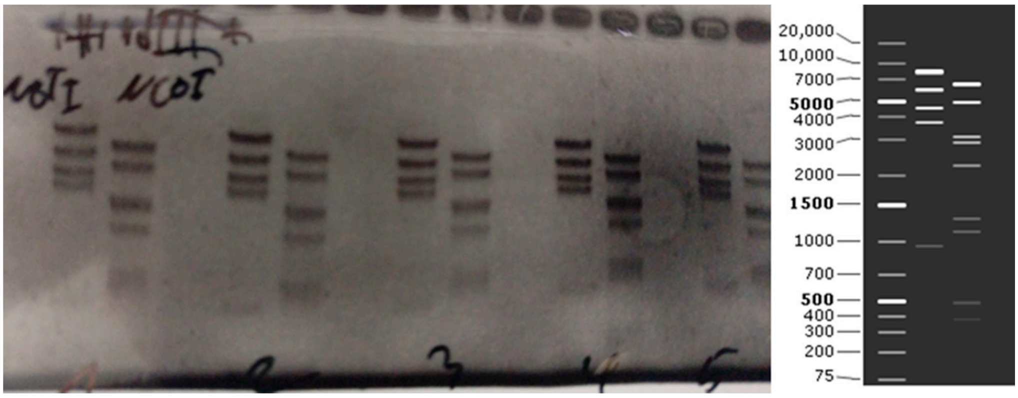

Figure S3: Test restriction of pCAP03-PUM with *Not*I/*Hin*dIII and *Nco*I/*Hin*dIII (left). *In silico* prediction of the restriction analysis (right).

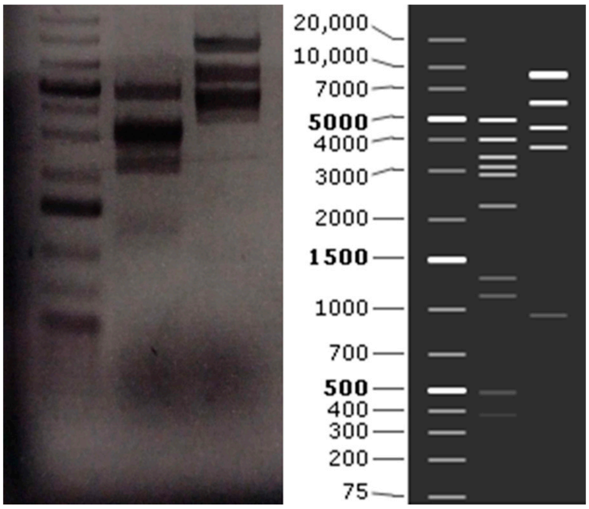

Table S1: Strains used in this study

Strain	Genotype	Reference
Streptomyces sp. DSM26212	WT	[1]
Streptomyces coelicolor M1146	Δact Δred Δcpk Δcda	[10]
E. coli Top10	F- mcrA Δ(mrr-hsdRMS-mcrBC) ϕ80lacZΔM15 ΔlacX74 nupG recA1 araD139 Δ(ara-leu)7697 galE15 galK16 rpsL(StrR) endA1 λ-	commercially available (invitrogen)
E. coli ET12567	dam-13::Tn9, dcm-6, hsdM	[17]
E. coli ET12567 + pUB307	dam-13::Tn9, dcm-6, hsdM + pUB307	[17]
E. coli BW25113 + pKD46	lacI+rrnBT14 ΔlacZWJ16 hsdR514 ΔaraBADAH33 ΔrhaBADLD78 rph-1 Δ(araB–D)567 Δ(rhaD–B)568 ΔlacZ4787(::rrnB-3) hsdR514 rph-1 + pKD46	[16]
E. coli XL1 Blue + pIJ773	recA1 endA1 gyrA96 thi-1 hsdR17 supE44 relA1 lac + pIJ773	[14]
E. coli XL1 Blue + pCAP03	recA1 endA1 gyrA96 thi-1 hsdR17 supE44 relA1 lac + pCAP03	[15]
E. coli XL1 Blue + pGEM-t easy-Apra-ermE*	recA1 endA1 gyrA96 thi-1 hsdR17 supE44 relA1 lac + pGEM-teasy-Apra-ermE*	this work
E. coli XL1 Blue + pGEM-t easy-Apra-tcp830	recA1 endA1 gyrA96 thi-1 hsdR17 supE44 relA1 lac + pGEM-teasy-Apra-tcp830	this work
E. coli Top10 + pCAP03-PUMΔHΔF	F- mcrA Δ(mrr-hsdRMS-mcrBC) ϕ80lacZΔM15 ΔlacX74 nupG recA1 araD139 Δ(ara-leu)7697 galE15 galK16 rpsL(StrR) endA1 λ- + pCAP03-PUMΔHΔF	this work
E. coli Top10 + pCAP03-PUMΔF	F- mcrA Δ(mrr-hsdRMS-mcrBC) ϕ80lacZΔM15 ΔlacX74 nupG recA1 araD139 Δ(ara-leu)7697 galE15 galK16 rpsL(StrR) endA1 λ- + pCAP03-PUMΔF	this work
E. coli Top10 + pCAP03-PUM	F- mcrA Δ(mrr-hsdRMS-mcrBC) ϕ80lacZΔM15 ΔlacX74 nupG recA1 araD139 Δ(ara-leu)7697 galE15 galK16 rpsL(StrR) endA1 λ- + pCAP03-PUM	this work
E. coli BW25113 + pKD46 + pCAP03-PUMΔHΔF	lacI+rrnBT14 ΔlacZWJ16 hsdR514 ΔaraBADAH33 ΔrhaBADLD78 rph-1 Δ(araB–D)567 Δ(rhaD–B)568 ΔlacZ4787(::rrnB-3) hsdR514 rph-1 + pKD46 + pCAP03-PUMΔHΔF	this work
E. coli BW25113 + pKD46 + pCAP03-PUMΔF	lacI+rrnBT14 ΔlacZWJ16 hsdR514 ΔaraBADAH33 ΔrhaBADLD78 rph-1 Δ(araB–D)567 Δ(rhaD–B)568 ΔlacZ4787(::rrnB-3) hsdR514 rph-1 + pKD46 + pCAP03-PUMΔF	this work
E. coli BW25113 + pKD46 + pCAP03-PUM	lacI+rrnBT14 ΔlacZWJ16 hsdR514 ΔaraBADAH33 ΔrhaBADLD78 rph-1 Δ(araB–D)567 Δ(rhaD–B)568 ΔlacZ4787(::rrnB-3) hsdR514 rph-1 + pKD46 + pCAP03-PUM	this work
E. coli BW25113 + pCAP03-PUMΔHΔF_ermE*	lacI+rrnBT14 ΔlacZWJ16 hsdR514 ΔaraBADAH33 ΔrhaBADLD78 rph-1 Δ(araB–D)567 Δ(rhaD–B)568 ΔlacZ4787(::rrnB-3)	this work

	hsdR514 rph-1- + pCAP03-PUMΔHΔF_ermE*	
E. coli BW25113 + pCAP03-PUMΔF_ermE*	lacI+rrnBT14 ΔlacZWJ16 hsdR514 ΔaraBADAH33 ΔrhaBADLD78 rph-1 Δ(araB–D)567 Δ(rhaD–B)568 ΔlacZ4787(::rrnB-3) hsdR514 rph-1- + pCAP03-PUMΔF_ermE*	this work
E. coli BW25113 + pCAP03-PUM_ermE*	lacI+rrnBT14 ΔlacZWJ16 hsdR514 ΔaraBADAH33 ΔrhaBADLD78 rph-1 Δ(araB–D)567 Δ(rhaD–B)568 ΔlacZ4787(::rrnB-3) hsdR514 rph-1- + pCAP03-PUM_ermE*	this work
E. coli BW25113 + pCAP03-PUMΔHΔF_tcp830	lacI+rrnBT14 ΔlacZWJ16 hsdR514 ΔaraBADAH33 ΔrhaBADLD78 rph-1 Δ(araB–D)567 Δ(rhaD–B)568 ΔlacZ4787(::rrnB-3) hsdR514 rph-1- + pCAP03-PUMΔHΔF_tcp830	this work
E. coli BW25113 + pCAP03-PUMΔF_tcp830	lacI+rrnBT14 ΔlacZWJ16 hsdR514 ΔaraBADAH33 ΔrhaBADLD78 rph-1 Δ(araB–D)567 Δ(rhaD–B)568 ΔlacZ4787(::rrnB-3) hsdR514 rph-1- + pCAP03-PUMΔF_tcp830	this work
E. coli BW25113 + pCAP03-PUM_tcp830	lacI+rrnBT14 ΔlacZWJ16 hsdR514 ΔaraBADAH33 ΔrhaBADLD78 rph-1 Δ(araB–D)567 Δ(rhaD–B)568 ΔlacZ4787(::rrnB-3) hsdR514 rph-1- + pCAP03-PUM_tcp830	this work
E. coli ET12567 + pCAP03	dam-13::Tn9, dcm-6, hsdM + pCAP03	this work
E. coli ET12567 + pCAP03-PUMΔHΔF_ermE*	dam-13::Tn9, dcm-6, hsdM + pCAP03-PUMΔH_ermE*	this work
E. coli ET12567 + pCAP03-PUMΔF_ermE*	dam-13::Tn9, dcm-6, hsdM + pCAP03-PUMΔF_ermE*	this work
E. coli ET12567 + pCAP03-PUM_ermE*	dam-13::Tn9, dcm-6, hsdM + pCAP03-PUM_ermE*	this work
E. coli ET12567 + pCAP03-PUMΔHΔF_tcp830	dam-13::Tn9, dcm-6, hsdM + pCAP03-PUMΔF_tcp830	this work
E. coli ET12567 + pCAP03-PUMΔF_tcp830	dam-13::Tn9, dcm-6, hsdM + pCAP03-PUMΔF_tcp830	this work
E. coli ET12567 + pCAP03-PUM_tcp830	dam-13::Tn9, dcm-6, hsdM + pCAP03-PUM_tcp830	this work
Streptomyces coelicolor M1146 pCAP03-PUMΔFΔH_ermE*	Δact Δred Δcpk Δcda + pCAP03-ermE*-pumB,D,G,E,I,J,K,L,M,N	this work
Streptomyces coelicolor M1146 pCAP03-PUMΔF_ermE*	Δact Δred Δcpk Δcda + pCAP03-ermE*-pumB,D,G,E,H,I,J,K,L,M,N	this work
Streptomyces coelicolor M1146 pCAP03-PUM_ermE*	Δact Δred Δcpk Δcda + pCAP03-ermE*-pumB,D,G,E,F,H,I,J,K,L,M,N	this work
Streptomyces coelicolor M1146 pCAP03-PUMΔFΔH_tcp830	Δact Δred Δcpk Δcda + pCAP03-tcp830-pumB,D,G,E,I,J,K,L,M,N	this work
Streptomyces coelicolor M1146 pCAP03-PUMΔF_tcp830	Δact Δred Δcpk Δcda + pCAP03-tcp830-pumB,D,G,E,H,I,J,K,L,M,N	this work
Streptomyces coelicolor M1146 pCAP03-PUM_tcp830	Δact Δred Δcpk Δcda + pCAP03-tcp830-pumB,D,G,E,F,H,I,J,K,L,M,N	this work

Table S2: Primers used in this study

Name	Sequence (5' -> 3')
pIJ773cass_f	ATTCCGGGGATCCGTCGACC
ermEp1	CCTCCCACCCGCTGGATCCTACCAACCGGCACGATTGTCCAGCCCACAACAGCATCGCGGTGCCACGT GTGGACCGCGTCGGTCAGATCCTCCCCGCA
ermEp2	TGCTGTTGTGGGCACAATCGTGCCGGTTGGTAGGATCCAGCGggtaggagg
tcp830	CCTCCCAGATCTCTATCACTGATAGGGATCCTACCACTATCAATGATAGAGTAGCCAACAGCTGTAGG CTGGAGCTGCTTC
pumB_f	CATGGTATAAATAGTGGCGTGGATAGATACCTACGAGC
pumB_r	GTGGTCACCCTCCTCATACCGCCTCCGTCTCCA
pumD_f	CGGTATGAGGAGGGTGACCACGTGACGGGCACC
pumD_r	CGATCATGCAGGCCGCCTCCTCAGGTCCATTGACTGAGAG
pumG_f	GGAGGCGGCCTGCATGATCGGCGGCATGTCGCT
pumG_r	GATGCCTCCACGTTTATCAGTCACAGGTCCGCAAGAGCCT
pumG+F_r	TGTAAAGGCCTCCGTCACAGGTCCGCAAGAGCCT
pumE_f	CTGATAAACGTGGAGGCATCATGTCGATTTCCCTGTCGTT
pumE_r	CAGAACTCCCTCCTCACTCGGATCCGTCCCGGC
pumEdH_r	GACGCATCGGTACTCCCTCCTCACTCGGATCCGTCCCGGC
pumF_f	CCTGTGACGGAGGCCTTTACAGTGTGGAACGTC
pumF_r	GATGCCTCCACGTTTATCAGTCATCGACCGCTCCGGGATC
pumI_f	GGAGGGAGTACCGATGCGTCAGGGCTTCGATGA
pumH_f	CCGAGTGAGGAGGGAGTTCTGGTGATCATTGAGGGC
pumJ_r	GGACAGCCTCCCGGGGACGCTCAGCGGCGGAGGACCAACT
pumK_f	GCGTCCCCGGGAGGCTGTCCATGGCGTTGCTGCTGCTCAA
pumN_r	TATGTAGCTTTCGACATATTAGGCCAACGGCCGGTAAC
pCAP03_1f	TGTCGAAAGCTACATATAAG
pCAP03_1r	AACTGTTCGCCAGGCTCAAG
pCAP03_2f	CTTGAGCCTGGCGAACAGTT
pCAP03_2r	GCCACTATTTATACCATGGG
Rec_uni_f	CCCTGTCGCCCTCCTATTGGCTTCCGGATTATCTTCTTGGCAGCTCACGGTAACTGATG
PUM_ermE*_rec	GCCAGTCGGCCGGCTCGTAGGTATCTATCCACGCCACTATCCTCCTACCCGCTGGATCCT
PUM_tcp830_rec	GCCAGTCGGCCGGCTCGTAGGTATCTATCCACGCCACTATCCTCCCAGATCTCTATCACT
Pum_screen	GCATGGGCGTTCATGCTGAC
ermE*_rectest	ATCTTGACGGCTGGCGAGAG
tcp830_rectest	CAGCTGTTGGCTACTCTATC
